# The Decrease in Human Endogenous Retrovirus-H Activity Runs in Parallel with Improvement in ADHD Symptoms in Patients Undergoing Methylphenidate Therapy

**DOI:** 10.3390/ijms19113286

**Published:** 2018-10-23

**Authors:** Cipriani Chiara, Pitzianti Maria Bernanda, Matteucci Claudia, D’Agati Elisa, Miele Martino Tony, Rapaccini Valentina, Grelli Sandro, Curatolo Paolo, Sinibaldi-Vallebona Paola, Pasini Augusto, Balestrieri Emanuela

**Affiliations:** 1 Department of Experimental Medicine and Surgery, University of Rome Tor Vergata, Via Montpellier 1, 00133 Rome, Italy; chiaracipriani88@gmail.com (C.C.); matteucci@med.uniroma2.it (M.C.); miele@med.uniroma2.it (M.M.T.); grelli@med.uniroma2.it (G.S.); sinibaldi-vallebona@med.uniroma2.it (S.-V.P.); 2 Child Neurology and Psychiatry Unit, Systems Medicine Department, University Hospital Tor Vergata, Viale Oxford 81, 00133 Rome, Italy; b.pitzianti@libero.it (P.M.B.); elisadagati@gmail.com (D.E.); rapaccinivalentina@gmail.com (R.V.); curatolo@uniroma2.it (C.P.); pasini@uniroma2.it (P.A.); 3 Unità Sanitaria Locale (USL) Umbria 2, Viale VIII Marzo, 05100 Terni, Italy; 4 Institute of Translational Pharmacology, National Research Council, Via Fosso del Cavaliere 100, 00133 Rome, Italy

**Keywords:** HERVs, HERV-H, ADHD, methylphenidate, neurodevelopmental disorders, environmental stimuli

## Abstract

Increasing scientific evidence demonstrated the deregulation of human endogenous retroviruses (HERVs) expression in complex diseases, such as cancer, autoimmune, psychiatric, and neurological disorders. The dynamic regulation of HERV activity and their responsiveness to a variety of environmental stimuli designate HERVs as genetic elements that could be modulated by drugs. Methylphenidate (MPH) is widely used in the treatment of attention deficit hyperactivity disorder (ADHD). The aim of this study was to evaluate the time course of human endogenous retrovirus H (HERV-H) expression in peripheral blood mononuclear cells (PBMCs) with respect to clinical response in ADHD patients undergoing MPH therapy. A fast reduction in HERV-H activity in ADHD patients undergoing MPH therapy was observed in parallel with an improvement in clinical symptoms. Moreover, when PBMCs from drug-naïve patients were cultured in vitro, HERV-H expression increased, while no changes in the expression levels were found in ADHD patients undergoing therapy. This suggests that MPH could affect the HERV-H activity and supports the hypothesis that high expression levels of HERV-H could be considered a distinctive trait of ADHD patients.

## 1. Introduction

Endogenous retroviruses are genetic elements present in the genomes of all vertebrates, including humans [1,2]. They are residual of ancestral infections of germ cells by exogenous viruses, which have been integrated as proviruses into the host genome and transmitted to subsequent generations in a Mendelian fashion [3,4,5].

During evolution, human endogenous retroviruses (HERVs) amplified and spread throughout the entire genome by repeated events of retrotransposition and/or reinfection [6]. Their integration into the genome alters the structure and/or the function of neighboring genes [7]. Currently, about 8% of the human genome consists of endogenous retroviral sequences [8]. Many cellular mechanisms have evolved to restrict HERVs’ intracellular ability to replicate and to express mRNAs and proteins, including deletion and recombination events, epigenetic mechanisms such as DNA methylation and chromatin remodeling, post-transcriptional processing, and RNA interference [9,10]. However, at least some members of the HERV groups are still transcriptionally active in a tissue-specific manner [11,12], maintaining open reading frames (ORFs) that potentially code for viral proteins [13].

HERVs have been mainly taken into account for their role in the molecular evolution of genomes [14]. However, in the last decades, several studies have underlined their involvement in the etiopathogenesis of complex diseases, such as cancer [15,16], autoimmune diseases [17], type 1 diabetes [18], and neurological and psychiatric disorders [19].

Peculiarly, numerous endogenous/exogenous factors lead to the activation of HERVs, including hormones [20], cytokines [21], cytotoxic chemicals/drugs [22,23], and interactions with microorganisms [24,25].

In addition, HERV expression can be modified by different types of drugs, such as DNA methyltransferase and histone deacetylase inhibitors [26,27], antiretroviral drugs [28,29,30], and neuroleptics and/or antidepressants (valproic acid, haloperidol, risperidone, clozapine) [22]. In agreement, in an earlier study using a murine model of autism we showed that valproic acid activates endogenous retroviruses expression in blood and brain tissue [31].

Among several HERV groups, copies of HERV-H have been found distributed throughout the entire human genome [32], with the majority of HERV-H elements showing large deletions in the *pol* region, lack of the entire *env* region, and rare full-length copies with intact ORFs [33]. The presence of HERV-H insertional polymorphisms in human genome supports the idea that this group is still active [34], contributing to pluripotency in human embryonic stem cells harboring binding sites of pluripotency transcription factors, such as NANOG, OCT4, and SOX2 [35]. Expression of several HERV groups, including HERV-H, has also been demonstrated in different types of cancer [36]. In patients with active multiple sclerosis, antibody reactivity towards HERV-H env and high expression of HERV-H env epitopes on B cells and monocytes have been found [37]. Among neurodevelopmental disorders, an increased transcriptional activity of HERV-H sequences has been found in patients with autism spectrum disorders (ASD) [38,39] and attention deficit hyperactivity disorder (ADHD) [40].

With an estimated prevalence of 5% in children and 2.5% in adults in the United States, ADHD is one of the most common neurodevelopmental disorders, which leads to persistent inattention, hyperactivity, and impulsivity [41]. Among a wide variety of pharmacological options available in ADHD treatment, the stimulant drug methylphenidate (MPH) is the most frequently prescribed in the treatment of children. It is believed that MPH increases the concentration of catecholamines, including dopamine and norepinephrine, in the synaptic cleft by blocking their reuptake [42,43]. MPH is thought to predominantly affect the dopaminergic system, and its action consists of blocking the reverse dopamine transporter (DAT) [44]. Magnetic resonance studies and/or positron emission tomography and genetic studies using molecular techniques have revealed that the ADHD neurobiological substratum consists of a dopaminergic system dysfunction and an alteration of cerebral networks involving the frontostriatal system [45,46,47,48]. Moreover, it has been shown that the attentional processes and the ability to inhibit impulsive responses are mediated by catecholaminergic neurotransmitters, such as dopamine and noradrenaline [49,50].

Several studies have shown that MPH is able to improve the core symptoms of ADHD [51,52], and the efficacy of pharmacological treatment has been demonstrated by improvements in a variety of social settings [53]. In our previous work, we described a high transcriptional activity of HERV-H in peripheral blood mononuclear cells (PBMCs) from 30 drug-naïve ADHD children compared to healthy controls, that correlated positively with the core symptoms of the disorder, suggesting HERV-H as a possible new molecular signature of the disease [40]. More recently, we demonstrated a significant reduction in HERV-H expression associated with improvement in ADHD symptoms in a 16-year-old ADHD patient after six months of MPH therapy [54]. On this basis, the aim of this study was to evaluate the time course of HERV-H expression with respect to clinical response in ADHD patients undergoing MPH therapy. For this purpose, HERV-H expression was analyzed in fresh- and in-vitro-stimulated PBMCs from drug-naïve ADHD patients after 1, 8, and 24 weeks of therapy.

## 2. Results

**Figure 1:**
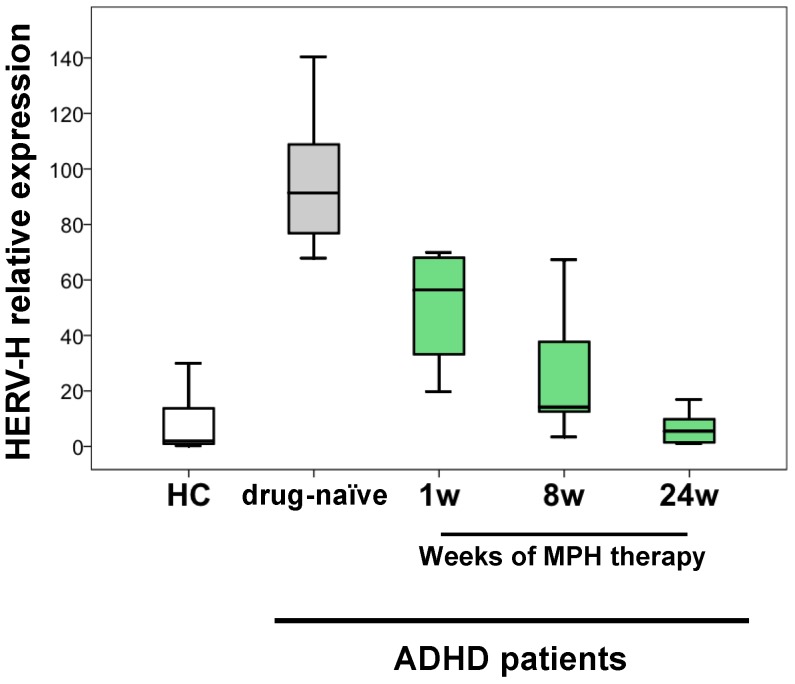
HERV-H expression in peripheral blood mononuclear cells (PBMCs) from attention deficit hyperactivity disorder (ADHD) patients at different times of methylphenidate (MPH) therapy. HERV-H relative expression was evaluated in fresh PBMCs from seven drug-naïve ADHD patients (grey box plot) and after 1, 8, and 24 weeks of MPH therapy (green box plots) and compared to that obtained in fresh PBMCs from 12 healthy controls (HC), age- and sex-matched (white box plot).

In order to evaluate the time course of HERV-H expression during MPH therapy, the transcriptional activity was evaluated in fresh PBMCs from drug-naïve ADHD patients (grey box plot in Figure 1) after 1, 8, and 24 weeks of MPH therapy (green box plots).

The transcriptional levels evaluated by real-time RT-PCR were compared to those in fresh PBMCs from age- and sex-matched healthy controls (HC) (white box plot). Before therapy, relative HERV-H expression was significantly higher in PBMCs from ADHD patients compared to HC (*p* < 0.001). As early as the first week of treatment, HERV-H relative expression significantly decreased (*p* = 0.012), and a further reduction was observed after eight weeks (*p* = 0.001) and 24 weeks (*p* = 0.001) of therapy. Notably, after 24 weeks of MPH therapy, HERV-H levels were comparable to those found in PBMCs from HC (*p* = 0.659).

The intensity and frequency of the core symptoms of ADHD were assessed with the long version of the Conners’ Parents Rating Scale-Revised questionnaire (CPRS-R). The CPRS-R was conducted at eight and 24 weeks after MPH treatment as any clinical response cannot be detected before that time [55,56]. Four clinical variables were considered: the Conners’ parent oppositional (CP-O), the Conners’ parent inattention (CP-I), the parent hyperactivity/impulsivity (CP-H), and the Conners’ parent ADHD-Index (CP-AI).

**Figure 2:**
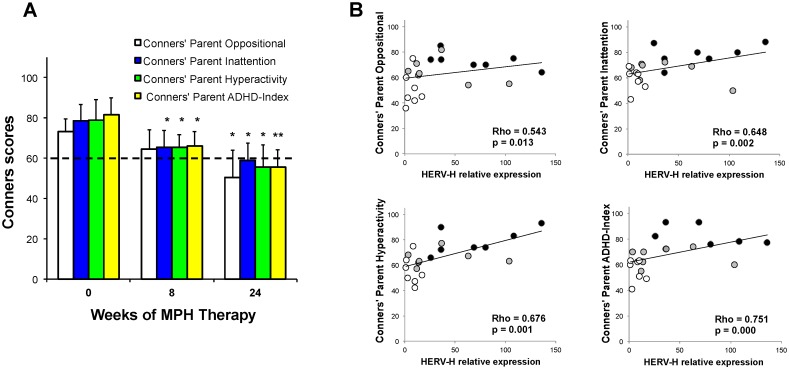
Clinical variables from Conners’ Parents Rating Scale-Revised (CPRS-R) and human endogenous retrovirus H (HERV-H) expression. (**A**) Mean values ± standard deviations (SD) of the four clinical variables (Conners’ parent oppositional, Conners’ parent inattention, Conners’ parent hyperactivity/impulsivity, and Conners’ parent ADHD-Index) in seven ADHD patients before the beginning of MPH therapy and after eight and 24 weeks. The dashed line represents the cut-off score. Single asterisk (*) indicates *p* values < 0.05 and double (**) indicates *p* values < 0.001. (**B**) HERV-H relative expression evaluated by real-time RT-PCR analysis plotted against the clinical variables. The patients are represented according to the time points by different colors: black for patients at the beginning of therapy, grey for patients analyzed after eight weeks of therapy, and white for patients after 24 weeks of therapy. Rho and *p* values for Spearman correlation analysis are shown.

In Figure 2, panel A represents the mean values ± standard deviations (SD) of the scores recorded before and during the MPH therapy.

A general trend of reduction for all the scores was observed in response to therapy. In particular, when compared to the scores values before therapy, a statistically significant decrease in the CP-I (*p* = 0.028), CP-H (*p* = 0.044), and CP-AI (*p* = 0.006) scores was observed after eight weeks of therapy. After 24 weeks of therapy, a significant decrease in the CP-I (*p* = 0.001), CP-H (*p* = 0.001), CP-O (*p* = 0.002) and a highly significant decrease in CP-AI (*p* < 0.001) was achieved.

As the decreasing trend observed in Conners’ scores (Figure 2, panel A) paralleled with HERV-H expression during therapy (Figure 1), to assess this association, we performed a Spearman correlation analysis between the expression of HERV-H and the values for the different clinical scores. The statistical analysis demonstrated a positive correlation between HERV-H relative expression and all the scores values (Figure 2, panel B): in particular CP-O (rho 0.543, *p* = 0.013), CP-I (rho 0.648, *p* = 0.002), CP-H (0.676, *p* = 0.001) and CP-AI (rho 0.751, *p* < 0.001), in ADHD patients before and during treatment.

**Figure 3:**
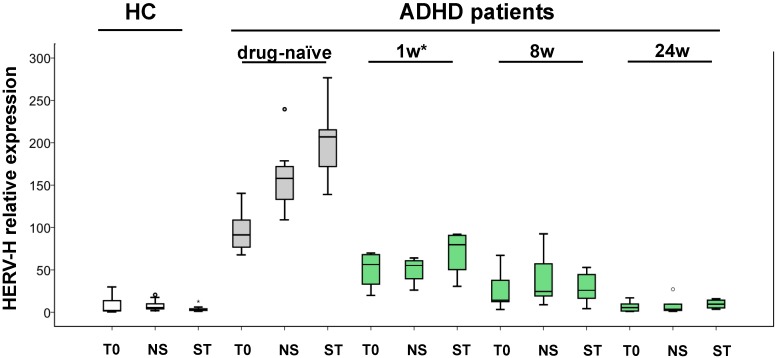
HERV-H expression in PBMCs from ADHD patients after in vitro culture. The relative expression of HERV-H was evaluated in fresh PBMCs (T0) and after 72 h in culture in absence (not stimulated, NS) or in presence of IL-2 and PHA (stimulated, ST). The levels were measured in seven drug-naïve ADHD patients (grey box plots) and during MPH therapy (green box plots) at 1, 8, and 24 weeks. The results were compared to those obtained in PBMCs from 12 healthy controls (HC) (white box plots), maintained in the same culture conditions (* weeks of therapy).

Finally, in order to consider cell responsiveness to the in vitro stimulation with IL-2 and PHA, HERV-H transcriptional activity was assessed in cultured PBMCs from ADHD patients. To this purpose, HERV-H relative expression was evaluated by real-time RT-PCR in fresh PBMCs (T0) and after 72 h of culture in absence (not stimulated, NS) or in presence of IL-2 and PHA (stimulated, ST) before and during MPH therapy. The HERV-H relative expression was also evaluated in PBMCs from HC maintained in the same culture conditions. In PBMCs from drug-naïve ADHD patients, HERV-H expression was significantly higher after 72 h of culture in both the conditions, i.e., in presence or not of IL-2/PHA, with respect to fresh PBMCs (*p* ≤ 0.004) (Figure 3, grey box plots). Conversely, when PBMCs from ADHD patients in therapy were cultured in vitro, no differences in HERV-H expression were found, either in presence or not of IL-2/PHA, at all the observation times (1, 8, and 24 weeks) after the beginning of therapy (Figure 3, green box plots). Likewise, the PBMCs from HC did not show any changes in HERV-H expression when maintained in culture (Figure 3, white box plots).

## 3. Discussion

Scientific reports support the involvement of HERV genetic elements in many complex human diseases, including neurological and psychiatric disorders [19]. Evidence of an association between HERV expression and neurodevelopmental diseases had also emerged from our previous published studies in which we demonstrated an increase in HERV-H transcriptional activity in PBMCs from ASD [38,39] and drug-naïve ADHD patients [40] compared to healthy controls, suggesting that HERVs could play a role in the etiology of these complex diseases. Moreover, HERV-H transcriptional activity correlated with inattention and hyperactivity symptoms in ADHD patients [40]. Interestingly, we had also described the reduction in HERV-H expression and the significant improvement in ADHD symptoms in PBMCs from an ADHD patient after 24 weeks of MPH treatment [54]. In agreement with our previous findings, the present study showed that the HERV-H expression was higher in drug-naïve ADHD patients compared to HC and was significantly reduced after 24 weeks of MPH treatment. These data further support our hypothesis that the transcriptional activation of this specific retroviral element might represent a molecular signature of the disorder.

Herein, we analyzed the time-course of HERV-H transcriptional activity in PBMCs from ADHD patients after 1, 8, and 24 weeks of MPH therapy, demonstrating that the expression of HERV-H significantly decreased after only one week. Interestingly, at this time of observation, no significant improvement of clinical symptoms by MPH treatment can be achieved [55,56]. Subsequent to the fast downregulation of HERV-H expression, a further decreasing trend was confirmed throughout the 24 weeks of therapy. Notably, at the endpoint of observation, HERV-H expression in treated ADHD patients reached levels comparable to those found in HC. The improvement of the clinical signs, as evidenced by the reduction in the CPRS-R scores during MPH treatment, proceeded in parallel with the decrease in HERV-H expression, and the statistical analysis demonstrated the correlation between the CPRS-R scores and HERV-H expression levels. All these data support the hypothesis that the deregulation of HERV-H expression is closely associated with the disorder.

Treatment with neuroleptics and/or antidepressants induces epigenetic modifications influencing gene expression [22,57,58]. By exploring the mechanism of action of MPH, the modulation of the expression of several genes has been demonstrated in animal models as well as in ADHD patients. In the striatum of MPH-treated rats, more than 700 genes were found upregulated [59]. These genes are involved in migration of immature neural/glial cells and/or growth of novel axons, active axonal myelination, upregulation of mature processes, and more enduring enhancement of neurobehavioral plasticity [59]. Recently, long non-coding RNAs (lncRNAs) signature in the prefrontal cortex of MPH-exposed rats was identified [60] and among the lncRNAs modulated by MPH, the MRAK081997 positively correlated with the dihydrofolatereductase gene, which may be involved in axon regeneration in rodents through DNA methylation [61]. Finally, a microarray analysis of patient-derived lymphoblastoid cells revealed that several genes were regulated by MPH treatment [62].

The responsiveness to environmental triggers designates HERVs as genetic elements that could be modulated by MPH treatment. Our thought is supported by evidence that HERV activity is modulated in response to a variety of environmental stimuli, including epigenetic drugs [63]. On the other hand, the HERV sequences spread in the genome may regulate the expression of neighboring genes [7,64]. Particularly, HERV-H, acting as promoter enhancer of nearby genes and functioning as lncRNAs, plays an important role in the pluripotency of human cells [35], and the aberrant HERV-H expression in embryonic stem cells and induced pluripotent stem cells determines the differentiation-defective phenotype in neural lineage [65,66,67]. We recently demonstrated in a valproic acid-induced mouse model of ASD that high expression of different murine ERVs and inflammatory mediators was related to autistic-like traits. Notably, we showed that the high levels of ERVs expression identified in brain were also revealed in blood tissue from the same mice, supporting the view that altered ERVs expression in the blood could be a reliable biomarker for brain atypical development [31].

Finally, herein we reported an increase in HERV-H expression in response to culture or stimulation in vitro (with IL-2 and PHA) of PBMCs from drug-naïve ADHD patients, which was in line with our previous findings in ASD patients [38]. Intriguingly, this intrinsic predisposition to express HERV-H, observed in PBMCs of drug-naïve patients, was lost early after MPH therapy, suggesting that the drug could directly or indirectly influence HERV-H activity. In addition, no changes in expression levels of HERV-H were observed in PBMCs from HC after culture or stimulation in vitro, supporting the hypothesis that the predisposition to express HERV-H could be considered as a distinctive trait of drug-naïve ADHD patients.

Although the present study provides preliminary data, we have highlighted for the first time the fast decrease in HERV-H activity after only one week of MPH treatment and how the further activity reduction runs in parallel with improvement in symptoms in ADHD patients undergoing therapy. MPH is the most frequently used drug in ADHD treatment, showing several favorable effects on symptoms; however, its use is associated with serious and nonserious adverse events, both in children and adolescents, with about 30% of patients not responding to the therapy [68,69,70]. In this context, it may be important to identify patients who are most susceptible to adverse events or are nonresponders in order to select patients to whom MPH treatment could exert major benefits. Future well-designed prospective studies will greatly help to candidate HERV-H as a predictive marker of the response to MPH therapy.

## 4. Materials and Methods

### 4.1. Participants

The study included 7 drug-naive ADHD patients, all males and aged between 7 and 17 years (median age 13) with IQ > 80, recruited among those attending the Child Neurology and Psychiatry Unit of “Tor Vergata” University Hospital of Rome (Table 1).

The patients were compared to 12 healthy controls (HC) of the same sex, aged between 7 and 17 years (median age 11), attending the outpatient facilities of the same hospital for routine control visits. None of them had a history of neurological or psychiatric disorders, learning disability, or infectious diseases.

All participants were of Caucasian origin without significant economic, social, and cultural differences.

At the time of onset of the study, no participants were taking medication known to affect the central nervous system.

The study was carried out following the rules of the Declaration of Helsinki of 1975 (revised in 2008); the University Hospital of “Tor Vergata” Ethics Committee approved the study, and all examinations were performed after receiving written informed consent of the parents.

### 4.2. Clinical Assessment

The diagnosis of ADHD was based on clinical assessment, observations of children, and interviews with parents and children, which were carried out by an experienced child psychiatrist. To make the diagnosis of ADHD, the long version of the Conners’ Parents Rating Scale-Revised (CPRS-R) was used, including four clinical variables: the Conners’ parent oppositional (CP-O), the Conners’ parent inattention (CP-I), the Conner’ parent hyperactivity/impulsivity (CP-H), and the Conners’ parent ADHD-Index (CP-AI) [71]. The CPRS-R was also conducted after eight and 24 weeks of MPH treatment, to evaluate the clinical response. The interview with the Schedule for Affective Disorders and Schizophrenia for School-Age Children—Present and Lifetime Version (K-SADS-PL) was used to exclude other psychiatric comorbidities in the ADHD group [72].

### 4.3. Pharmacological Intervention

The planned treating schedule required the administration of MPH at the dose of 0.3 mg/kg/die for 1 week to subsequently reach the entire dose (0.5 ÷ 0.8 mg/kg/die). The immediate release formulation was used during the MPH titration phase. Once the absence of adverse effects was tested, the patients were treated with modified-release MPH because it allows once-daily dosing and therefore guarantees better compliance with the drug’s intake.

### 4.4. Samples Preparation and RT-PCR Analysis

PBMCs were separated by density gradient centrifugation (Lympholyte-H, Merck Darmstadt, Germany) from both ADHD patients and healthy controls. PBMCs were collected immediately after separation (fresh PBMCs) or cultured in RPMI 1640 medium (Merck, Darmstadt, Germany) supplemented with 12% fetal bovine serum (Life Technologies, Carlsbad, CA, USA), 2 mM glutamine (Merck, Darmstadt, Germany), 50 U/mL penicillin, 50 U/mL streptomycin (Merck, Darmstadt, Germany) at 37 °C under 5% CO2, without any stimulation (condition termed “not stimulated”, NS) or in presence of human recombinant interleukin-2 (IL-2), 20 U/mL (Chiron corporation, Emeryville, CA, USA) and T-lymphocyte-specific mitogen phytohemagglutinin (PHA), 2 µg/mL (Merck, Darmstadt, Germany) (condition called “stimulated”, ST).

HERV-H activity was evaluated both in fresh and cultured PBMCs of drug-naïve patients and after 1, 8, and 24 weeks of MPH therapy. The expression levels of the *env* sequence from HERV-H were quantitatively assessed by real-time RT-PCR, as previously described [38]. Briefly, 250 ng of DNase-treated RNA from PBMCs of ADHD patients and HC subjects were reverse-transcribed and amplified using primers specific for HERV-H and the housekeeping gene glucoronidase beta (*GUSB*) using SYBR Green chemistry. Each experiment was completed with a melting curve analysis to confirm the specificity of amplification and the relative expression was calculated as 2^−[ΔCt(sample) − ΔCt(calibrator)]^, where Δ Ct(sample) = [Ct(HERV-H *env*) − Ct(*GUSB*)],and Δ Ct(calibrator) was the mean of ΔCT of data obtained from fresh PBMCs of HC individuals. Real-time RT-PCR results were represented by box plots.

### 4.5. Statistical Analysis

The Mann–Whitney test was used to compare HERV-H relative expression between ADHD and HC groups, within ADHD patients at different times of therapy, and in all the conditions analyzed. The ANOVA analysis of variance and post-hoc Bonferroni tests were used to determine changes in Conners’ parent scores (CP-O, CP-I, CP-H, and CP-AI) during the treatment. To determine any correlation between HERV-H relative expression and core symptoms (or scores value), the Spearman’s rho correlation coefficient was calculated. Statistical analyses were carried out using Statistical Package for the Social Sciences (SPSS) software version 23.0 (SPSS Inc., Chicago, IL, USA). Statistical significant comparisons were considered when *p* < 0.050.

## 5. Conclusions

Growing evidence supports the role of HERVs in the onset and/or progression of several complex diseases, such as cancer, autoimmunity, neurological, and psychiatric disorders. Spatial and temporal fine-tuning mechanisms regulate HERV expression, and numerous endogenous/exogenous factors influence their activity, as the main common feature of HERVs is the responsiveness to environmental stimuli. Several drugs seem to affect HERV expression, candidating HERVs as predictive markers for the response to therapy, especially for those disorders where none of the available clinical parameters can discriminate a non-response as a priori or an early response after the beginning of therapy.

## Figures and Tables

**Table 1:** Demographic information of individuals included in the study.

	ADHD Patients (*n* = 7)	Healthy Controls (*n* = 12)	*p* Value
Gender	males	males	1
Median age (range) years	13 (7–17)	11 (7–17)	0.249
